# Objective assessment of shared plate eating using a wearable camera in urban and rural households in Ghana

**DOI:** 10.3389/fnut.2024.1428771

**Published:** 2024-09-12

**Authors:** Christabel A. Domfe, Megan A. McCrory, Edward Sazonov, Tonmoy Ghosh, Viprav Raju, Gary Frost, Matilda Steiner-Asiedu, Mingui Sun, Wenyan Jia, Tom Baranowski, Benny Lo, Alex K. Anderson

**Affiliations:** ^1^Department of Nutritional Sciences, University of Georgia, Athens, GA, United States; ^2^Department of Health Sciences, Boston University, Boston, MA, United States; ^3^Department of Electrical and Computer Engineering, University of Alabama, Tuscaloosa, AL, United States; ^4^Section for Nutrition Research, Department of Metabolism, Digestion and Reproduction, Imperial College, London, United Kingdom; ^5^Department of Nutrition and Food Science, University of Ghana, Accra, Ghana; ^6^Department of Neurosurgery, University of Pittsburgh, Pittsburgh, PA, United States; ^7^Department of Electrical and Computer Engineering, University of Pittsburgh, Pittsburgh, PA, United States; ^8^USDA/ARS Children’s Nutrition Research Center, Department of Pediatrics, Baylor College of Medicine, Houston, TX, United States; ^9^Hamlyn Center, Imperial College, London, United Kingdom

**Keywords:** shared plate eating, low-and middle-income countries, dietary assessment, technology, wearable camera, households, urban/rural

## Abstract

**Background:**

Shared plate eating (SPE), defined as two or more individuals eating directly from the same plate or bowl, is a common household food consumption practice in many Low- and Middle-Income Countries (LMICs). Examination of household engagement in SPE remains largely unexplored, highlighting a gap in research when interpreting dietary information obtained from these settings. The dearth of research into SPE can be attributed to the inherent limitations of traditional dietary assessment methods which constrain their usability in settings where SPE is common.

**Objective:**

In this expository narrative, we describe what SPE is when it is practiced in an LMIC such as Ghana; and also compare the frequency of SPE versus individual plate eating (IPE) by different household members in rural and urban households using a wearable camera (Automatic Ingestion Monitor version 2: AIM-2).

**Methods:**

Purposive convenience sampling was employed to recruit and enroll 30 households each from an urban and a rural community (*n* = 60 households) in Ghana. The AIM-2 was worn on eyeglass frames for 3 days by selected household members. The AIM-2, when worn, automatically collects images to capture food consumption in participants’ environments, thus enabling passive capture of household SPE dynamics.

**Results:**

A higher percentage of SPE occasions was observed for rural (96.7%) compared to urban (36.7%) households (*p* < 0.001). Common SPE dynamics included only adults sharing, adults and children sharing, only children sharing, and non-household member participation in SPE.

**Conclusion:**

The wearable camera captured eating dynamics within households that would have likely been missed or altered by traditional dietary assessment methods. Obtaining reliable and accurate data is crucial for assessing dietary intake in settings where SPE is a norm.

## Introduction

Cultural practices dictate how food is acquired, prepared, shared, and consumed at the household level. Household food consumption occurs in unique fashions in many Low- and Middle-Income Countries (LMICs), particularly communal eating, in which groups of people huddle and share food (1). Several benefits have been attributed to this practice, including improving the sensorial qualities of the food (2) and cordiality within groups, creating a sense of community (3). In many such societies, communal eating is embraced whereas individual eating is shunned (4).

Shared Plate Eating (SPE) is a food consumption practice where food is eaten directly from a single bowl or plate by two or more people, irrespective of their number or ages. SPE is commonly practiced in LMICs (1), particularly Asian (3, 5, 6) and African countries (7). Various scenarios typically play out during SPE depending on the specific population, the number of people, their ages, genders, and the types and amounts of food being shared, etc., which may affect what and how much is consumed by each participating member (6). A study among Negev Bedouin Arabs in Southern Israel showed that 88% of the participants engaged in SPE, indicating it was the usual practice in that population, though it was more common in rural than urban areas (5). SPE was less common among rural Nepalese children, with just 25% of all eating occasions within the day being SPE (6). In resource-limited settings, SPE influences the sharers to become intensely competitive to get a greater portion of the food (8). In instances where children share with adults, children may be at an eating disadvantage, unless there is a mother (or motherly figure) also partaking, wherein the child may benefit from being fed or being prompted to eat more (6). In Kalama, a peri-urban town in Egypt, the entire family sat around a table to share the food, which almost always featured bread in addition to whatever was being served (7). The bread served as a scoop for the food as cutlery was hardly used. School-aged boys were typically exempted from SPE as they were served their food on individual plates (IPE).

Individual dietary intake assessment from SPE is challenging (1). During SPE, sharers tend to eat different components of a meal, especially when multiple foods make up the meal, e.g., Ghanaian foods such as *waakye* (rice and beans) mixed with tomato stew, hot pepper sauce, spaghetti, roasted grated cassava (*gari*), fried fish, and vegetables; or *fufu* (pounded cassava/yam and plantain) served with multiple animal source foods and a soup base (9). Assessment challenges include variations in food contents within each handful when using hands to eat (as is usually the case) and whether every food component is eaten by each person sharing (1). Additional challenges not specific to SPE include variability in ingredients and recipes for preparing similar foods across households within the same community, and sometimes highly unreliable nationally representative food composition databases in countries where SPE is common (8, 10).

Furthermore, context-appropriate dietary assessment tools are lacking, likely because commonly used tools and traditional methods for dietary assessment were developed for use in High-Income Countries (HICs) where SPE is rarely practiced (1, 5). For example, the 24-h dietary recall (24hdr) method has been widely used for individual intake assessment from SPE (1). However, since the 24hdr was not intended for assessing individual intake from SPE, quality dietary information oftentimes cannot be obtained (5). Female household members, especially mothers, have been observed to typically report and quantify dietary intake from SPE (7). In situations where surrogate respondents have to quantify individual intake from SPE, they would need to know beforehand individual portion sizes consumed by sharers of a plate; how to mentally convert consumed portions into standard quantities; and consciously pay attention to what parts of the meal and amounts are consumed (11) to provide an accurate report, which is difficult to achieve.

Valid methods for assessing dietary intake in populations where SPE is common are necessary to establish suitable dietary intake guidelines, accurately assess nutritional status, and establish diet-disease relationships (12). A suggested approach to determine individual intake from SPE in Egypt was to first obtain quantitative data on the group partaking in the SPE activity (e.g., husband and wife, parents and children, children, etc.) and then obtain qualitative data based on the culture of the people (7). Challenges associated with this method may include non-household member participation and variations in the number of individuals present at the start and end of consumption. This can occur due to individuals joining or leaving during the consumption period, leading to discrepancies in the number of participants engaged in an SPE episode. There have been calls for technologies that address the challenges of commonly used traditional dietary assessment methods (13). In response, numerous dietary assessment tools and methods have been generated (14). In LMICs, directly recording dietary intake information electronically via mobile devices such as tablets has been recommended to potentially save money and time (15). Automatic dietary monitoring, a method that collects and processes dietary information without the active involvement of the participant, has addressed the challenges of estimating the timing of intake, identifying the food/drink consumed, and estimating the portion consumed (16). Camera technology has been suggested, but the feasibility needs to be tested before adoption (1).

Studies examining the usual dynamics that typically feature SPE; and the frequency of SPE in rural compared to urban households in LMICs are currently lacking in the literature, especially those using new technologies. To bridge this gap, we examined SPE dynamics in urban and rural households in Ghana by different family members using a wearable camera for intake assessment. Exploration of this household eating dynamic can inform nutrition interventionists and policymakers about intrahousehold food distribution and consumption practices that may tend to perpetuate the double burden of malnutrition and micronutrient deficiencies within LMICs. In addition, published literature on SPE occasions shows an inconsistent use of terminologies that makes reconciliation of findings challenging, i.e., SPE vs. commensality vs. communal eating vs. common plate eating, etc. To help establish the use of consistent terminology, we specify what SPE is—food is eaten directly from a single bowl or plate by two or more people—when it is mentioned and practiced within an LMIC such as Ghana.

## Methods

### Study design

The present study was part of a larger cross-sectional community-based project validating innovative passive dietary assessment tools, whose protocol was described previously (17). Data were collected in households from two communities (urban vs. rural) in Ghana, chosen to reflect the diversity of food-related behaviors of Ghanaians. This study was conducted in Ghana because of the need for valuable insights into household food consumption practices that have the potential to perpetuate the double burden of malnutrition. In the Greater Accra Region, the University of Ghana Junior Staff Residence was selected to represent urban households. A community in the Eastern Region, *Asaase Kokoo* was selected to represent rural households. Purposive convenience sampling was used to include households in which both parents (mother and father) lived together with a child under 5 years (C-U5) and/or an adolescent and in which all eligible household members partook in household eating occasions. Sixty (60) households were recruited, 30 each from the urban and rural communities.

The AIM-2 (13, 14) was the innovative technology-based means of dietary intake assessment that is reported here. It was worn by most recruited participants within each household, i.e., mother, father, and index child—i.e., the child under 5 and/or adolescent. The eligibility criteria for inclusion was to capture the diversity of food sharing and eating practices within households with children of different ages. While some households had more than three/four members who met the inclusion criteria, the main validation study only focused on these four (mother, father, child under 5 and/or adolescent). We used data collected during the field validation phase of the main project to examine SPE. Prior to the field validation phase, preliminary data were obtained during the feasibility phase of the study (18). The AIM-2 was used to capture the food intake of household members in their natural environments, without prescriptions as to how to go about their day-to-day activities.

Ethical approval was obtained from the Human Subject Institutional Review Boards of The University of Georgia (STUDY00006121) and Noguchi Memorial Institute of Medical Research, University of Ghana (#-046/18-19). Mothers and fathers provided consent by signing or thumbprinting, while the adolescents assented after the study protocol was explained to them and their questions were answered.

### Data collection

To assess the habitual dietary intake of the household, dietary data were collected over 3 days: 2 weekdays (WD1, WD2) and 1 weekend day (WE). On days of data collection, two field research assistants arrived at the homes of the participants just around the time household members arose from bed (around 6:30 am) and left the home environment when the household was done with all food-related activities for the day (around 6–7 pm in the rural community and 8 pm in the urban community). The times were informed by data from an earlier formative study (19). The RAs performed additional roles such as weighing food and assisting participants to set up the device(s) for use each morning. Specifically, regarding the AIM-2, they ensured that the devices were worn during food consumption. The RAs also remained around the participants’ homes to troubleshoot any issues with the devices. Participants wore AIM-2 (camera affixed to eyeglasses) throughout the day (from wake time to after the last eating occasion of the day), in order not to miss any food consumption activity. Participants were instructed to take the device off when they required privacy (e.g., bathroom use). Children under 5 who could wear the AIM-2 device did so only during meal times. After eating, the device was removed to prevent them from playing with it or causing any damage.

A standard household characteristics questionnaire was completed by the household head and/or the primary caregiver within a 3-day period. The questionnaire included items on household size, educational attainment, prioritization of certain members during food sharing, and whether households engaged in SPE.

### AIM technology (device and software)

**Figure 1:**
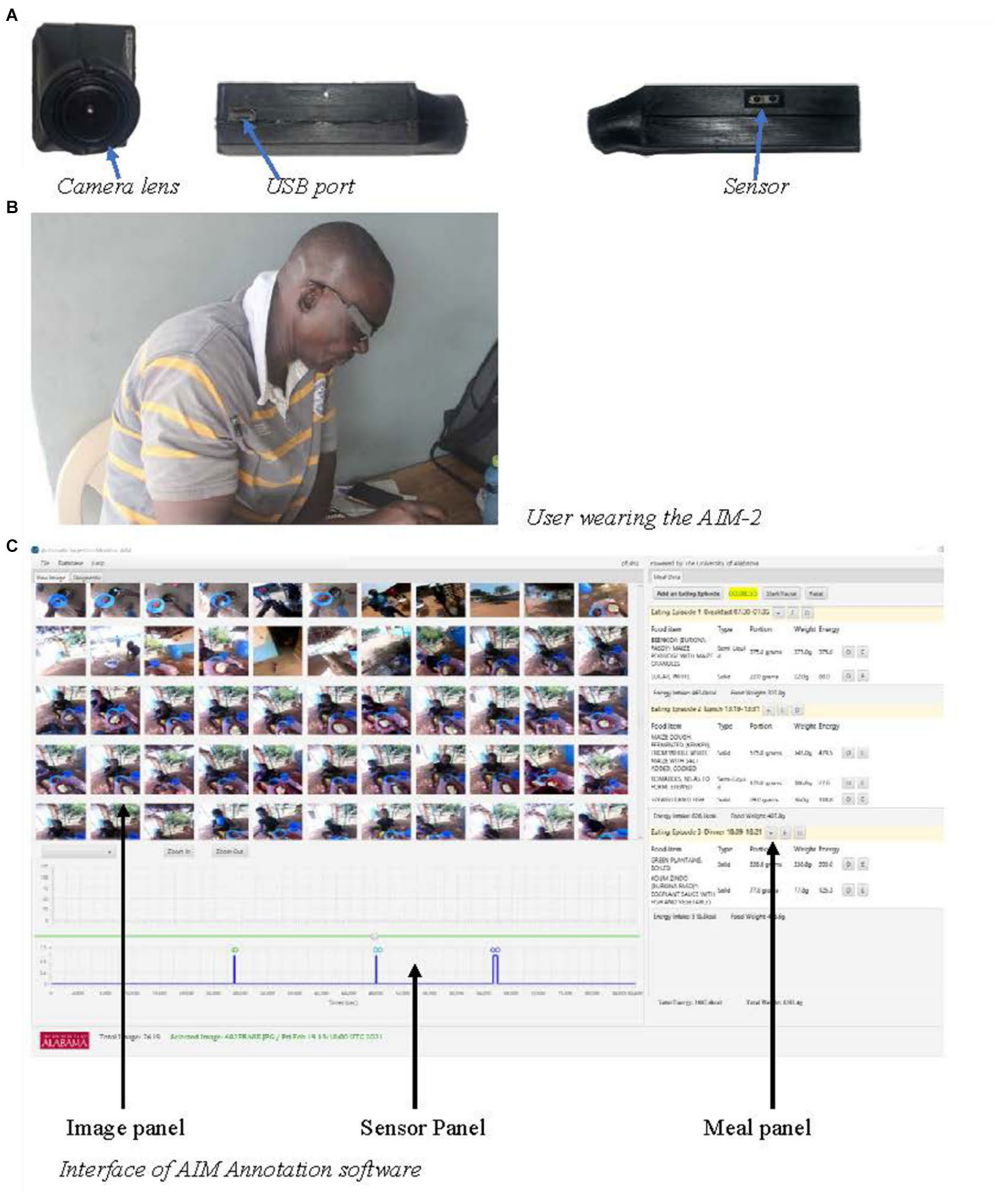
Components of the AIM-2.

The AIM-2 is a passive wearable sensor technology for objectively assessing food and beverage intake (20) (Figure 1). The device has a camera lens, USB port, and optical sensor (Figure 1A), attached to an eyeglass (Figure 1B). Internally (not shown) there is a 3-D accelerometer that detects head movement. The USB port enables device charging and data download. The camera lens enables image capture of everything in front of the participant within the range of image capture (focused at 20 cm to infinity) (21). The AIM-2 was programmed to take a single picture every 15 s. The AIM-2 does not record videos or audio. The optical sensor and accelerometer together detected food and beverage consumption by monitoring the contraction of the temporalis muscle and head movement. The accelerometer also allowed for detecting compliance with wearing the device (22).

After each day’s data collection, image and sensor data were downloaded from the device and uploaded to a secure cloud data storage space or an external drive. The device was charged overnight to last the entire next day’s worth of data collection. The field coordinator was responsible for downloading the data from the device, uploading to the storage cloud, and charging the device. The uploaded data were then processed into an accessible format that could be uploaded into the AIM Image Annotation Software version 4.5 (21) for nutritional analysis by a trained analyst. The software has three interface sections: image, sensor, and meal data panels (Figure 1C). The image panel enables a trained analyst to see the dynamics during food consumption or meal sequence, i.e., from the start through to the end of food consumption, the number of people engaged in the eating activity, and the appearance and disappearance of food. The sensor panel displays sensor peaks that correspond to food consumption episodes (but were not used for this analysis). The meal data panel displays the output of food consumed, the quantity consumed, and the weight of leftovers if any after the trained analyst estimates portion size. As part of the validation study, the research assistants recorded the weights of all ingredients used in the recipes, as well as the weights of the foods consumed and any leftovers. The results from these weighed food records will be reported elsewhere. This software ultimately enables the quantification of energy and nutrients from consumed foods using the programmed food composition database(s) (21). The observed frequencies of SPE vs. IPE are reported. Information on energy and nutrients will be reported elsewhere.

After an SPE episode was identified by reviewing the images, the analyst noted the following in a spreadsheet external to the software: the number and composition of people who shared the food, food appearance and disappearance as shown by hand-to-mouth movements, the dynamics playing out, i.e., noting if all members were feeding themselves or if some were being fed by others, when a composite meal (multiple food items constituting a meal) was shared, determination of how much of each component is eaten by the sharers. When there was at least one eating occasion where two or more people shared food over the 3 days, the household was determined to have engaged in SPE. Episodes not designated as SPE were deemed individual plate eating (IPE). Though a standard protocol for image annotation and a definition of SPE were developed and used by the nutrition team in charge of the image annotation, every SPE eating occasion featured different dynamics necessitating judgment calls to be made. For example, how much of each food component was consumed by sharers, and if additional servings of food were added to shared plate/bowl.

Essentially, the images captured by the AIM-2 provided enough detail about the type of food consumed, the number of people involved in the SPE, and the varying consumption dynamics that played out during the eating episode.

### Statistical analysis

We used IBM SPSS Statistics 27 for analysis. ANOVA was used to assess age differences between mothers and fathers and between urban and rural households. Descriptive statistics were calculated and reported as frequencies and percentages for categorical variables. Fischer’s Exact tests were performed to compare the frequencies of SPE and IPE between urban and rural households, by household member. Using Bonferroni’s correction, a *p*-value of ≤0.025 (i.e., 0.05/2) was used to determine statistical significance when comparing the frequencies of SPE and IPE due to comparisons between two factors: location and household members. A *p*-value of ≤0.05 was used to determine the statistical significance of the household characteristics.

## Results

### Household characteristics questionnaire data

Rural and urban household characteristics were comparable for sex of the household head, ethnicity, education level, and prioritizing some household members when sharing food, as no statistically significant differences were found (Table 1). On average, urban participants were older than their rural counterparts, and the fathers were older than the mothers irrespective of location. Statistically significant differences were noted in mealtimes where household members typically eat from a shared plate based on questionnaire data, with 47% of rural households indicating that dinner was more commonly consumed as SPE, while 50% of urban households reported lunch as more frequently eaten as SPE (*p* = 0.029). Additionally, 60% of rural households vs. 26% of urban households reported adults and children eating from the same plate (*p* = 0.035). Furthermore, 47% of rural households indicated eating outside in the open, whereas 50% of urban households ate anywhere within the house (*p* = 0.029) in response to where food is usually eaten in the home. More than three-quarters of the rural households answered in the affirmative when asked about engaging in SPE compared to about half of urban households (*p* = 0.033).

**Table 1:** Urban/rural comparison of household characteristics.

Variable	Rural ( $\bar{x}$ ± SD)	Urban ( $\bar{x}$ ± SD)	*p*-value	
Age (years)				
Fathers	39.6 ± 12.4	44.9 ± 8.7	**0.008**	
Mothers	34.5 ± 10.7	44.4 ± 7.4	**<0.001**	
	***n* (%)**	***n* (%)**	**Total *n* (%)**	***p*-value**
Sex of respondent				
*Male*	13 (43.3)	2 (6.7)	15 (25)	**0.002**
*Female*	17 (56.7)	28 (93.3)	45 (75)	
Sex of household head				
*Male*	28 (93.3)	30 (100)	58 (96.7)	0.492
*Female*	2 (6.7)	0 (0)	2 (3.3)	
Ethnicity				
*Akan*	10 (33.3)	14 (46.7)	24 (40)	0.292
*Ga*	0 (0)	2 (6.7)	2 (3.3)	
*Guan*	5 (16.7)	2 (6.7)	7 (11.7)	
*Ewe*	14 (46.7)	10 (33.3)	24 (40)	
*Other*	1 (3.3)	2 (6.7)	3 (5)	
Respondent’s educational level				
*Basic*	9 (30)	6 (20)	15 (25)	
*Junior high*	16 (53.3)	13 (43.3)	29 (48.3)	0.362
*Senior high*	3 (10)	6 (20)	9 (15)	
*Tertiary*	2 (6.7)	5 (16.7)	7 (11.7)	
How many people live in the household?				
*≤4*	14 (46.7)	4 (13.3)	18 (30)	**0.014**
*5*	6 (20)	13 (43.3)	19 (31.7)	
*≥6*	10 (33.3)	13 (43.3)	23 (38.3)	
Do you own your own home?				
*Yes*	18 (60)	1 (3.3)	19 (31.7)	**0.001**
*No*	12 (40)	29 (96.7)	41 (68.3)	
Do some members of your household get priority in eating food?				
*Yes*	10 (33.3)	12 (40)	22 (36.7)	0.789
*No*	20 (66.7)	18 (60)	38 (63.3)	
Does your household eat from a shared plate?				
*Yes*	23 (76.7)	14 (46.7)	37 (61.7)	**0.033**
*No*	7 (23.3)	16 (53.3)	23 (38.3)	
Which mealtimes does your household usually eat from a shared plate?				
*Breakfast*	3 (10)	7 (23.3)	10 (16.7)	**0.029**
*Lunch*	13 (43.3)	15 (50)	28 (46.7)	
*Supper/dinner*	14 (46.7)	5 (16.7)	19 (31.7)	
*Other*	0 (0)	3 (10)	3 (5)	
Who in the household eats from a shared plate?				
*Adult only share*	5 (16.7)	7 (25.9)	12 (21.1)	**0.035**
*Children only share*	1 (3.3)	0 (0)	1 (1.8)	
*Both adult and children share*	18 (60)	7 (25.9)	25 (43.9)	
*Other*	6 (20)	13 (48.1)	19 (33.3)	
Where in the household is food usually eaten?				
Dining/designated room	3 (10)	7 (23.3)	10 (16.7)	
Anywhere within the house	13 (43.3)	15 (50)	28 (46.7)	
Outside the house	14 (46.7)	5 (16.7)	19 (31.7)	**0.029**
Other	0 (0)	3 (10)	3 (5)	

Age: *p*-values were obtained for rural vs urban fathers and rural vs urban mothers using ANOVA, while descriptive statistics were calculated and reported as frequencies and percentages (categorical variables). Bold values are significant at *p* ≤ 0.05.

### Rural/urban comparison of shared vs. individual plate eating with AIM-2

Examination of AIM-2 images showed that SPE was a common household food consumption practice, especially within rural households where nearly all households (96.7%) compared to about one-third of urban households (36.7%) engaged in any SPE (*p* < 0.001). Comparing the frequency of SPE and IPE across the 3 days of data collection between urban and rural households (Table 2), rural engagement in SPE was more common and frequent compared to urban households, particularly for dinner meals. Irrespective of the location, breakfast was more often consumed as IPE. Lunch was more likely to be consumed as SPE for rural mothers and fathers on the weekend day. Also, snack consumption was more likely to be consumed as IPE than SPE for both urban and rural locations. Irrespective of household location, no statistically significant differences in the frequencies of SPE vs. IPE were observed for all the eating occasions, except for snack consumption on WD1 for rural (*p* = 0.025) and WE for urban (*p* < 0.001) participants.

**Table 2:** Comparison of individual and shared plate eating occasion frequencies between urban and rural households.

Eating occasion	WD1			WD2			WE		
	Mothers Rural	Urban	*p*	Rural	Urban	*p*	Rural	Urban	*p*
	*n* (%)	*n* (%)		*n* (%)	*n* (%)		*n* (%)	*n* (%)	
Mothers									
Breakfast									
*SPE*	11 (39)	0 (0)	**0.002**	16 (70)	1 (4)	**<0.001**	6 (30)	1 (5)	0.091
*IPE*	17 (61)	20 (100)		7 (30)	24 (96)		14 (70)	18 (95)	
Lunch									
*SPE*	8 (62)	6 (30)	0.148	4 (36)	5 (36)	1.000	13 (72)	3 (14)	**<0.001**
*IPE*	5 (39)	14 (70)		7 (64)	9 (64)		5 (28)	18 (86)	
Dinner									
*SPE*	22 (82)	6 (29)	**<0.001**	19 (70)	7 (29)	**0.005**	18 (72)	4 (22)	**0.002**
*IPE*	5 (19)	15 (71)		8 (30)	17 (71)		7 (28)	14 (78)	
Snack									
*SPE*	0 (0)	1 (11)	0.450	0 (0)	1 (6)	0.424	2 (18)	0 (0)	0.485
*IPE*	11 (100)	8 (89)		6 (100)	15 (94)		9 (82)	8 (100)	
Fathers									
Breakfast									
*SPE*	12 (52)	0 (0)	**<0.001**	12 (60)	1 (4)		7 (39)	0 (0)	
*IPE*	11 (48)	18 (100)		8 (40)	22 (96)	**<0.001**	11 (61)	15 (100)	**0.009**
Lunch									
*SPE*	8 (44)	1 (7)	0.044	7 (64)	1 (13)	0.059	9 (64)	2 (13)	**0.008**
*IPE*	10 (56)	13 (93)		4 (36)	7 (83)		5 (36)	13 (87)	
Dinner									
*SPE*	18 (69)	5 (23)	**0.002**	17 (63)	2 (9)	**<0.001**	16 (70)	2 (13)	**<0.001**
*IPE*	8 (31)	17 (77)		10 (37)	20 (91)		7 (30)	14 (87)	
Snack									
*SPE*	1 (11)	0 (0)	1.000	1 (11)	0 (0)	1.000	2 (17)	0 (0)	0.559
*IPE*	8 (89)	6 (100)		8 (89)	7 (100)		10 (83)	5 (100)	
Adolescents									
Breakfast									
*SPE*	4 (44)	0 (0)	**0.012**	5 (56)	0 (0)	**0.002**	4 (57)	0 (0)	**0.003**
*IPE*	5 (56)	15 (100)		4 (44)	16 (100)		3 (43)	17 (100)	
Lunch									
*SPE*	3 (60)	0 (0)	0.035	3 (60)	1 (14)	0.222	3 (43)	0 (0)	0.043
*IPE*	2 (40)	8 (100)		2 (40)	6 (86)		4 (57)	11 (100)	
Dinner									
*SPE*	7 (64)	2 (13)	**0.014**	6 (50)	2 (12)	0.038	7 (58)	2 (15)	0.041
*IPE*	4 (36)	13 (87)		6 (50)	15 (88)		5 (42)	11 (85)	
Snack									
*SPE*	1 (25)	0 (0)	1.000	3 (38)	0 (0)	0.231	2 (29)	0 (0)	0.462
*IPE*	3 (75)	3 (100)		5 (63)	5 (100)		5 (71)	6 (100)	
Children under-5									
Breakfast									
*SPE*	5 (42)	0 (0)	0.045	6 (50)	1 (10)	0.074	3 (27)	1 (9)	0.586
Children under-5									
*IPE*	7 (58)	9 (100)		6 (50)	9 (90)		8 (73)	10 (91)	
Lunch									
*SPE*	3 (43)	4 (40)	1.000	3 (50)	4 (44)	1.000	5 (63)	1 (11)	0.050
*IPE*	4 (57)	6 (60)		3 (50)	5 (56)		3 (38)	8 (89)	
Dinner									
*SPE*	10 (83)	4 (36)	0.036	8 (62)	3 (30)	0.214	4 (50)	3 (27)	0.377
*IPE*	2 (17)	7 (64)		5 (39)	7 (70)		4 (50)	8 (73)	
Snack									
*SPE*	4 (57)	2 (33)	0.592	3 (38)	0 (0)	0.209	1 (13)	0 (0)	1.000
*IPE*	3 (43)	4 (67)		5 (63)	6 (100)		7 (88)	6 (100)	

*n, number of people; %, percentage; WD1, weekday 1; WD2, weekday 2; WE, weekend day; SPE, shared plate eating; IPE, individual plate eating. Fischer’s Exact tests were performed to compare the frequency of shared and individual plate eating between and within urban and rural households.*

Using Bonferroni’s correction, a *p*-value of ≤ 0.025 (i.e., 0.05/2) was used to determine statistical significance. Bold values are significant at *p* ≤0.025.

The mean ages of children under 5 were 2.1 ± 1.0 years in rural households and 3.6 ± 0.7 years in urban households. For adolescents, the mean ages were 14.4 ± 2.6 years in rural households and 14.9 ± 2.7 years in urban households. In total, there were 12 children < 5 and 14 adolescents in rural households, and 17 children < 5 and 19 adolescents in urban households.

### Notable SPE scenarios observed

Regardless of the area of residence, it was commonplace for couples only, the couple and younger children, children only, and mothers and their children to eat together from the same bowl/plate. SPE was observed for breakfast, lunch, dinner, and snacks across both areas of residences. No particular order was observed in food sharing. Described below are some of the common dynamics that featured SPE occasions.

**Figure 2:**
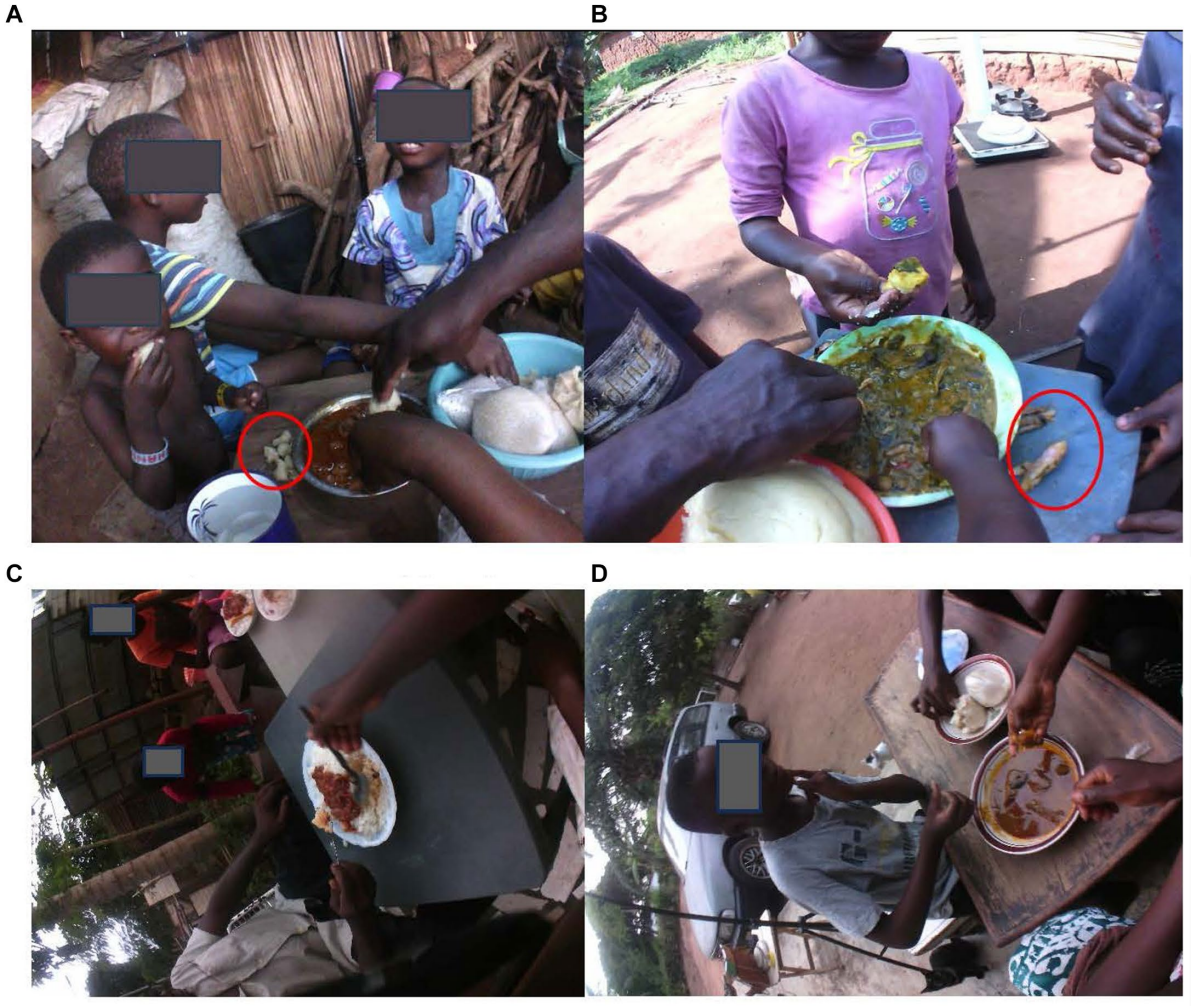
Rural and urban SPE dynamics as captured by the AIM-2. **(A)** Starchy component placed in front of young child. **(B)** Protein portion dished out. Rural SPE dynamics involving young children and adults. **(C)** Couple sharing a plate of food. **(D)** Mother shares with adolescent children. Urban SPE dynamics (no component of food dished out).

1. Adults-only sharing: This dynamic was especially observed for the rural mother–father dyads. Notably during dinner, rural couples would sit to share food from the same bowl. Also, it was commonplace for the men in the rural community to take a break from work (be it farming, brewing alcoholic beverages, or construction) and sit together to share food from the same bowl for lunch. In these scenarios, individual intake assessment was possible for the individuals wearing the AIM-2 by looking at the captured images.
2. Adults and children sharing: This was common in urban and rural households. An SPE episode could feature a mother and/or father with older and/or younger children. There were instances when younger children benefitted in such a scenario by being fed by one of the parents. This was not always observed. In some instances, what they managed to grasp and put in their mouths were the only portions consumed. In one household, the staple and not the accompanying soup base or protein was what was mainly consumed by the young child sharing with older siblings and adults. Interestingly, an insightful practice was also observed when younger children shared with adults in some rural households. The protein portion and sometimes the starchy staple were dished out of the main bowl and placed on the table directly in front of the younger child (Figure 2). This was not observed in urban households.
3. Children [siblings] sharing: In some households, it was common for siblings to share a bowl/plate of food. Occasionally, the mother would divide the protein portion among the siblings before they began eating. At other times, an older sibling sharing the food would take on this responsibility.
4. Non-household member participation: Non-household member participation was observed within rural households in particular, where friends or extended family members were welcome to join a family SPE occasion.

### Infrequent scenarios observed include

1. Everyone shared the main staple, while two different accompanying stew or soup bases were served in separate bowls and consumed individually.
2. An adult started the meal and, after they had their fill, the remaining portions were shared among a group of children.
3. SPE during snack consumption, though rare, took various forms such as more than one person sharing a can or bottle of soda, breaking pieces from the same loaf of bread, or sharing a packet of cookies or crackers.

## Discussion

This is the first study to assess household SPE dynamics using wearable camera technology. As in many other LMICs, the SPE dynamic in Ghanaian households was a common feature of food consumption. However, many additional interesting and important findings were observed as enabled by the wearable camera. First, SPE was more common in rural compared to urban households. Second, there was great variation in SPE dynamics among household members at the household level but at the meal level, we found that SPE was more common during dinner meals for rural households than in urban households. Additionally, breakfast meals and snacks were more often consumed as IPE rather than SPE for both urban and rural participants. Third, there was a mismatch between the respondents’ self-reported answers about household members engaging in SPE and what was observed. Finally, examination of the image sequences within meals enabled the analyst to observe the intricacies of SPE dynamics, such as who was sharing, the number of people sharing, and the foods shared, all of which would be difficult or impossible to assess using traditional dietary recall assessment methods. These results highlight the advantages of using newer image-based technologies, such as passive image capture from wearable cameras, for assessing dietary intake in LMIC where SPE is commonplace.

A high frequency of SPE was observed in rural locations in Ghana, and a more moderate frequency in urban locations, which was consistent with findings in Southern Israel among Negev Bedouin Arabs conducted using a 24hrdr more than a decade ago (5). The rural Nepal study that used the direct observation method had a tendency to alter the usual intake with the presence of research staff (6). These two studies exemplify different methods previously used to report SPE frequency, both with potential drawbacks that can impact accuracy such as incorrect recall, or interference by research staff. We also assessed the frequency of SPE as self-reported by participants via questionnaire, with the results showing that 20% fewer rural households and 10% more urban households reported SPE as common in their homes compared to the SPE frequencies assessed by the AIM-2. The difference in reported versus observed amounts of SPE supports the importance of an objective tool for dietary intake assessment. Within the rural households, the difference may have been an attempt to impress research staff as being modern and not engaging in traditional consumption practices. Reliance on interview data only in the household characteristics questionnaire would have been biased in capturing the true occurrence of SPE.

An important finding in our study was the many and varied consumption dynamics during SPE, exemplifying the different ways in which SPE occurred in different households, many of which cannot be quantitatively captured. These dynamics emphasize the relevance of the AIM technology for individual intake assessment from SPE and not a reliance on recall methods—where these particular situations could easily be forgotten, or even the weighed food record—as individual portions consumed from a shared plate currently cannot be weighed. In another study, a 360^°^ camera with a deep learning technique was used to estimate individual intake from food-sharing scenarios through facial detection, food recognition, and detection of hand movement (23). However, food sharing in that study was not from a single bowl or plate and did not occur in the natural environment of the participants.

The wearable camera technology enabled observing insightful practices during SPE, e.g., the protein portion and/or starchy staple were dished from the bowl onto the table for younger children, and non-household members joined halfway through a meal. The objectivity of the AIM-2 minimizes the risk of misreporting that often occurs with reliance on people’s memories. In a narrative review of SPE assessment, Burrows and colleagues highlighted the capacity of technological innovations to address the dietary assessment challenges faced when assessing individual intakes from SPE (1). The AIM technology, comprising AIM-2 and the annotation software, enabled the analyst to identify SPE episodes and the different consumption dynamics associated with the practice. The images clearly showed the number of people involved, food appearance and disappearance, plate or bowl-to-mouth hand movements, the start and end of food consumption, etc. The captured images facilitate a clearer picture of within-household food distribution patterns during SPE episodes. SPE is a common aspect of food consumption traditions in Ghana, especially in rural areas. Understanding food consumption traditions has the potential to highlight the strengths and inadequacies of dietary/nutrient intake within households and provide an exposition of the double burden of malnutrition within households. The high frequency of SPE occasions observed and the apparent dearth of research in LMICs particularly in Africa highlights a major limitation when interpreting dietary information gathered from this region. When SPE is not assessed by an objective measure (non-self report) in LMICs, investigators need to note the lack of capture of SPE as a limitation, given how common SPE is.

It is worth noting that, when SPE is practiced in LMICs such as Ghana, the various forms in which this eating dynamic plays out are different from commensality or communal eating where food is consumed in the presence of others (3), and common-plate eating as described among the Negev Bedouin population in southern Israel where one main dish is served on a platter and the second dish is dipped into or scooped by a piece of flatbread (5). Due to these variations, our study findings may not be generalizable to all settings.

## Study strengths and limitations

The main strength of our study was the use of a wearable camera for objective assessment of eating activities, representing an advance over traditional dietary assessment methods’ reliance on participants’ memory and literacy. Additionally, participants wore the device throughout the day to automatically capture all eating occasions as they occurred without input required by the users. Other strengths were that multiple family members in households from both urban and rural communities wore the device for 3 days (2 weekdays and 1 weekend day), enabling the capture of normal day-to-day variability in intake. The study also had limitations, including a small sample size which was due to budget considerations, reducing our ability to detect differences and relationships. However, collection of data from 60 households (3–4 individuals per household, i.e., mothers, fathers, child under 5 years, and/or an adolescent), 3 days each, provided over 540 days of image data and will be an invaluable resource for future studies involving secondary analyses. Also, even though the participants were encouraged to go about their day-to-day activities as they typically might, the presence of research staff around the home environment may have induced some alteration(s) in participants’ food behavior(s). Also, consistent picture-taking throughout the day might have posed privacy concerns; however, all non-food-related pictures will be deleted after the completion of the data analysis.

## Conclusion

The wearable camera technology captured images to objectively examine household food consumption dynamics that may perpetuate household undernutrition. The high frequency of SPE occasions observed in the present study, and the apparent dearth of research in LMICs particularly in Africa highlights a major limitation when interpreting dietary information gathered from this region. When SPE is not assessed by objective measure in LMICs, investigators need to note the lack of capture of SPE as a limitation, given how common SPE is. This recommendation is proposed because knowing the dynamics of within-household food consumption practices, such as SPE could be important for answering questions relating to household double burden of malnutrition, which is common in many LMICs including Ghana.

SPE remains largely unexplored despite it being a common household food consumption practice in LMICs, especially in rural households. When SPE is practiced in Ghana, the food is eaten from a single bowl or plate (or staple in one and stew/soup base in another) by two or more people, regardless of their ages and number. Several dynamics typically occur that necessitate the use of innovative technologies to capture individual intake for dietary assessment. The use of a wearable camera technology that passively captures food intake enabled a clear depiction of SPE dynamics within the households without a reliance on memory or cognition as would have been required if a traditional dietary method had been used. Further studies are underway in our research group to assess energy and nutrient intake from SPE and its validity using wearable cameras.

Dietary assessment in LMICs continues to be described as burdensome to the respondent and interviewer, producing poor quality information, and resulting in inaccuracies (15, 24, 25). Technological advances in dietary data collection and analysis offer promise for improvement. There’s a potential for cost-savings, and reduction in biases, and the passive participation of respondents addresses the literacy and memory requirements associated with the weighed food record and 24-h dietary recall (16, 25). The feasibility of using automatic dietary monitoring during SPE needs to be further established in future studies.

## Data Availability

The original contributions presented in the study are included in the article/supplementary material, further inquiries can be directed to the corresponding author.
